# Thermally Induced Creep and Viscoelastic Behavior of Copper Micropillar Arrays

**DOI:** 10.1002/advs.202519178

**Published:** 2026-02-17

**Authors:** Miao Wang, Jihua Zhang, Libin Gao, Hongwei Chen, Wenbo Luo, Wenlei Li, Mingcheng Chen, Mengru Li, Dongbin Wang, Shuang Li, Ting Liu, Xingzhou Cai, Yong Li, Bin Peng, Wanli Zhang

**Affiliations:** ^1^ State Key Laboratory of Electronic Thin Films and Integrated Devices University of Electronic Science and Technology of China Chengdu China; ^2^ Research and Development Department 3D Chips (Guangdong) Technology Co., Ltd Dongguan China; ^3^ Research and Development Department Chengdu Micro‐Technology Co. Ltd Chengdu China

**Keywords:** copper micropillar arrays, creep behavior, microstructure evolution, nano‐indentation, thermal conductivity performance

## Abstract

Copper micropillar arrays integrated in microchannel structures promise superior heat dissipation, yet their long‐term reliability can be limited by creep deformation under thermomechanical constraints. Here, we fabricate Cu micropillar arrays (∼50 µm in diameter and ∼300 µm in height) via electrodeposition within through‐glass vias (TGVs) and elucidate how heat treatment tailors creep, viscoelastic response, and deformation mechanisms. Nanoindentation creep combined with EBSD and TEM reveals the transition: 200°C promotes grain‐boundary‐mediated deformation with the largest creep displacement (101 nm) and the smallest activation volume (0.64 nm^3^), whereas 300°C increases dislocation density and produces dislocation pile‐ups/networks that pin grain boundaries, reducing creep displacement to 42 nm, raising the stress exponent to 3.76, and the activation volume recovers to 1.49 nm^3^. Viscoelasticity is quantified with a generalized Kelvin model and retardation spectrum analysis, which shows suppressed relaxation activity after 300°C treatment. Thermal tests on arrays with varying pitch indicate that 70 µm spacing yields the most pronounced monitored‐surface temperature rise (46.5°C) under identical heating, implying enhanced effective heat transport through the micropillar network. These results establish a microstructure–mechanism–property framework to guide the design of Cu micropillar arrays that balance manufacturability, creep reliability, and thermal performance for microscale liquid‐cooling.

## Graphical abstract

Copper micropillar arrays are fabricated by electrodeposition within through‐glass‐via, and their thermally induced time‐dependent deformation is quantified. Nanoindentation creep, EBSD, and TEM reveal a heat‐treatment‐driven transition between grain‐boundary‐mediated deformation and dislocation‐dominated creep, while Kelvin modeling captures viscoelastic relaxation.

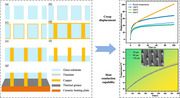

## 1 Introduction

In recent years, with the rapid development of Artificial Intelligence (AI), big data, and cloud computing, high‐performance computing chips (such as AI accelerators and GPUs), serving as the core hardware support, have seen continuous increases in both transistor density and operating frequency [1, 2]. While these advances enable exponential growth in computing capability, they also lead to a dramatic rise in heat flux density. If the generated heat cannot be dissipated in a timely and effective manner, it will result in decreased device performance, a shortened lifespan, and may even cause safety accidents [3, 4, 5, 6]. Among various heat transfer methods, convective heat dissipation has become a core consideration in the thermal design of electronic equipment due to its high controllability and efficiency. Accordingly, various active cooling technologies have been developed. Among them, liquid cooling technology, with its heat dissipation capacity far surpassing that of air cooling and its relatively quiet operation, has become an indispensable cooling solution for data centers and high‐performance computing [7, 8, 9, 10]. Its fundamental principle is to utilize the high specific heat capacity and thermal conductivity of liquids, using a pump to drive the circulation of coolant to carry away heat. However, in conventional liquid cooling systems—such as immersion cooling and cold plates—the heat exchange process typically occurs at a relatively macroscopic scale, with limited proximity between the coolant and the heat source and relatively simple flow channel geometries, thereby constraining further enhancement of heat transfer efficiency.

Recently, microchannel structures with micropillar arrays have shown great potential in achieving high‐efficiency heat dissipation by significantly increasing the heat exchange area and enhancing fluid disturbance to improve cooling efficiency [11, 12]. For example, leveraging the excellent thermal conductivity and the relatively low cost of aluminum, Fok et al. [13] used Selective Laser Melting (SLM) to integrate micropin fin arrays into aluminum substrates and investigated the heat dissipation capabilities of different pin fin geometries, finding that a novel airfoil‐shaped pin fin exhibited the best heat transfer performance at a Reynolds number (Re) of 1000. Yang et al. [14] studied square aluminum pin fins fabricated using CNC machining, discovering that the maximum surface heat transfer coefficient was achieved with a pin fin size of 100 µm, providing guidance for the design of phase‐change heat sinks such as heat pipes, 3D vapor chambers (3D‐VC), and microchannels. In addition, silicon‐based micropillar arrays fabricated through etching processes have been widely explored due to their excellent mechanical stability and compatibility with semiconductor manufacturing, making them suitable for chip‐level thermal management applications [15]. Li et al. [16] successfully fabricated a wick microstructure with micrometer‐scale copper pin fin arrays using ion etching, achieving up to a 75% improvement in the overall heat transfer coefficient compared to solid‐wall parallel microchannels.

Despite these advances, existing fabrication methods for micropillar or pin‐fin arrays still suffer from notable limitations. Techniques such as SLM and CNC machining are generally restricted to millimeter‐scale feature sizes and limited machining precision, which are unfavorable for the further miniaturization and uniformity required in advanced microchannel heat sinks [17]. Ion etching processes, although capable of producing finer structures, involve high fabrication costs and limited scalability, making them unsuitable for low‐cost, large‐scale manufacturing. Therefore, there is a strong demand for a fabrication strategy that enables high‐precision, cost‐effective, and scalable manufacturing of microscale pin‐fin arrays, while maintaining compatibility with advanced electronic packaging and microfluidic integration.

In response to these challenges, a fabrication approach combining laser induction, wet etching, and through‐glass via (TGV) electrochemical deposition has been developed to construct microfluidic heat dissipation structures integrated with microscale pin‐fin arrays on glass substrates [18, 19]. In this process, copper is selected as the pin‐fin material due to its unparalleled thermal conductivity (approximately 400 W/m·K), excellent electrochemical deposition characteristics, and high compatibility with micro‐ and nano‐fabrication techniques such as electroplating and wet etching. By filling TGVs with electrodeposited copper, dense and well‐defined copper micropillar arrays can be formed, enabling the construction of complex microfluidic channels with a large specific surface area. This composite structure effectively combines efficient heat conduction through copper micropillars with enhanced convective heat transfer provided by the microfluidic flow network.

In practical operation, however, copper micropillar arrays are subjected not only to thermal loads during operation but also to potential cyclic loads from fluid impact or stress induced by the constraints of the microfluidic channel structure [20, 21]. Given that copper intrinsically exhibits relatively low creep resistance—especially at elevated temperatures—long‐term exposure to sustained stress may lead to creep deformation of the micropillars [22, 23]. Such deformation can alter the pillar geometry, reduce flow channel patency, and ultimately degrade the overall heat dissipation performance. Therefore, ensuring the creep resistance of copper micropillars is a critical issue for their practical application in microchannel heat sinks.

Microstructural control is a common strategy for improving creep performance. It involves optimizing the material's microstructure, such as introducing strengthening phases, refining grains, or forming specific interface structures, to significantly enhance its service stability. For instance, researchers have successfully enhanced the creep resistance of copper and its alloys through various methods. Mahmudi et al. [24] prepared a Cu‐Cr alloy via a casting method and found that in situ‐formed Cr precipitates can effectively impede dislocation motion, thereby significantly enhancing the alloy's creep resistance. Similarly, Cai et al. [25] reported that introducing high‐density dislocations and microstrain into pure copper via cold rolling can also effectively increase its threshold stress and improve creep resistance. However, these strengthening strategies, which rely on alloying or severe macroscopic mechanical strain, are not applicable to electrodeposited copper micropillars. Wu et al. [26] investigated the effect of different current densities on the creep performance of electrodeposited twinned copper films, finding that as the current density increased from 40 ASD to 60 ASD, the creep displacement also showed an increasing trend, indicating a decrease in creep resistance. Singh et al. [27] studied the use of different additives to modulate twin spacing and their effect on creep displacement. Nevertheless, copper micropillars are typically fabricated at much lower current densities (below 2 ASD) with carefully tailored additive systems, resulting in microstructures that differ fundamentally from those of electrodeposited thin films. Considering the significant difference in thermal expansion coefficients between copper and glass, the microscopic strain generated when the copper column is heated can be utilized to improve its creep resistance. In this context, heat treatment, as a classic and highly controllable method for microstructural modification, offers a new pathway for optimizing the creep performance of copper micropillars.

In this study, copper micropillar arrays were successfully fabricated with varying pitches as the research subject. The creep behavior of the micropillars under different heat treatment parameters was analyzed through nanoindentation testing. Combined with the results from electron backscatter diffraction (EBSD) analysis and transmission electron microscope (TEM) analysis, the intrinsic relationship between their creep properties and microstructure was systematically investigated. The influence of different pitches of copper micropillar arrays on their thermal conductivity at room temperature was also explored. This research provides a process basis and data support for the fabrication of low‐cost and highly reliable copper micropillar arrays for microchannel heat dissipation applications.

## 2 Results and Discussion

### 2.1 Morphological Analysis of Copper Micropillars

**FIGURE 1:**
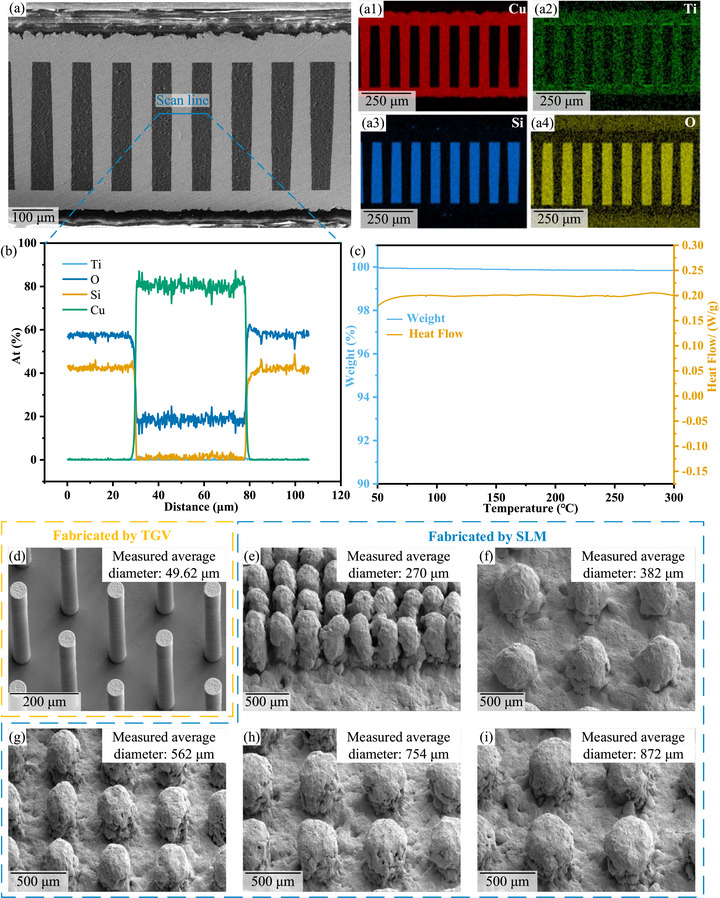
(a) The cross‐sectional SEM image of the copper micropillar sample after heat treatment at 300°C, (a1–a4) Corresponding energy‐dispersive X‐ray spectroscopy (EDS) elemental maps, (b) EDS line scan across the glass/copper interface, (c) TGA and DSC curve of test samples as the temperature changes, (d) The SEM image of copper micropillar with designed diameter of 50 µm fabricated by the TGV process, SEM images of copper micropillars with designed diameters of 100 µm (e), 300 µm (f), 500 µm (g), 600 µm (h), and 700 µm (i) fabricated by the SLM process. The corresponding measured diameter values are shown in the upper right corners.

Figures 1a and 1(a1–a4) show the cross‐sectional scanning electron microscopy (SEM) image and corresponding energy‐dispersive X‐ray spectroscopy (EDS) elemental maps, respectively, of the copper micropillar sample after heat treatment at 300°C. The results reveal a sharp and defect‐free interface between glass and copper, with no observable voids or cracks. An EDS line scan across the glass–copper interface (Figure 1b) demonstrates a distinctly abrupt transition in the concentration profiles of copper (Cu) and silicon (Si). Specifically, within the copper micropillar region, a strong Cu signal is detected along with a detectable amount of oxygen, likely originating from residual electroplating additives. The Cu signal drops rapidly to background levels on one side of the interface, while the Si signal rises sharply on the adjacent side. No significant interdiffusion or gradual compositional transition zone is observed between the two elements. In summary, the interface between copper micropillars and glass is not only morphologically sharp and well‐defined but also exhibits excellent thermo‐chemical stability, with no significant interfacial reaction observed even after heat treatment at 300°C.

To further evaluate the thermal stability of the interface during heating, thermogravimetric analysis (TGA) and differential scanning calorimetry (DSC) were performed, as shown in Figure 1c. The DSC curve exhibits no significant endothermic or exothermic peaks over the entire temperature range, indicating the absence of noticeable thermal events—such as phase transitions, chemical reactions, or interfacial compound formation—during heating. Concurrently, the TGA results show that the sample mass decreased only slightly from 100% to 99.85%, corresponding to a minimal mass loss of approximately 0.15%. This minor loss is attributed to the desorption of surface‐adsorbed moisture or trace organic residues, rather than decomposition or volatilization of the bulk materials. These thermal analysis results are highly consistent with the EDS line scan observations at the Cu/glass interface: the lack of distinct thermal effects and the negligible mass change corroborate the high thermal‐chemical stability of the Cu/glass interface during heating, with no evidence of significant elemental interdiffusion, chemical reaction, or interfacial degradation. This further explains the sharp boundary between Cu and Si observed in the EDS line scan—no pronounced thermally activated interfacial reactions occurred to blur the compositional transition. In summary, the copper micropillars and glass substrate not only form a morphologically sharp interface but also exhibit excellent thermo‐chemical stability under elevated temperatures.

A comparative surface morphology analysis of copper micropillar pin fins fabricated by through‐glass via (TGV) technology and those produced by selective laser melting (SLM) was conducted using SEM, as shown in Figure 1d–i. In this study, copper micropillars with different design diameters were fabricated using an SLM system equipped with a 532 nm single‐mode green fiber laser. The results indicate that, for the SLM process, the actual dimensions of the copper micropillars are significantly larger than the designed values. Specifically, when fabricating micropillars with a target diameter of 100 µm, severe adhesion (bridging) between adjacent features occurred, and the fabrication accuracy was very low. The surfaces of the resulting pin fins exhibited pronounced melt‐solidification characteristics, including obvious balling, adhered unmelted powder particles, and interlayer stair‐stepping effects. These defects led to high overall surface roughness, poorly defined geometric profiles, and limited dimensional precision. In contrast, the copper micropillar pin fins fabricated by the TGV process demonstrated excellent surface smoothness and structural fidelity, featuring continuous and smooth sidewalls, sharp edges, and minimal deviation in critical dimensions—highlighting the significant advantage of micro/nanofabrication techniques in the high‐precision formation of 3D metallic microstructures. These morphological differences stem from the fundamental nature of the two processes: SLM, as an additive manufacturing method, is inherently constrained by laser melt‐pool dynamics, powder bed uniformity, and thermal accumulation effects, making surface roughness and geometric distortion difficult to avoid. In comparison, the TGV process, integrating laser‐assisted via formation and electrodeposition, offers micron‐level pattern control, enabling the fabrication of high‐conformality, low‐roughness micro pin fins with superior dimensional accuracy.

### 2.2 Creep Behavior Analysis

Due to the micron‐scale dimensions of copper micropillars, conventional mechanical testing methods—such as tensile, compressive, and shear tests—are no longer suitable. Nanoindentation, by contrast, enables the measurement of mechanical properties at the micro‐ or even nanoscale with high repeatability, offering a significant advantage over traditional techniques. The nanoindentation theory established by Oliver and Pharr is widely recognized as an effective approach for determining the hardness (H) and Young's modulus (E) of both bulk and thin‐film materials [28, 29, 30]. Based on this theoretical framework, the present chapter employs nanoindentation to investigate the creep mechanical behavior of copper micropillars.

**FIGURE 2:**
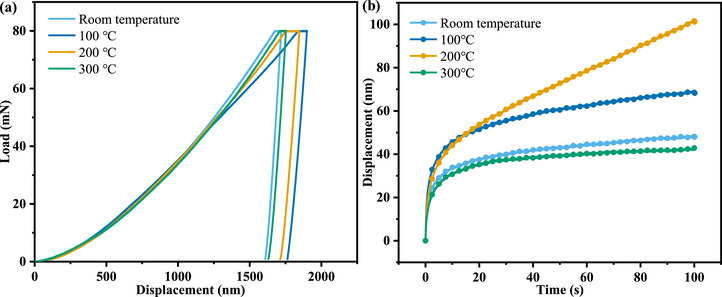
The load‐displacement curves (a) and displacement‐time curves (b) of copper micropillars.

Figure 2a presents the load (P) versus displacement (h) curves of copper micropillars before and after heat treatment. Prior to reaching the holding load, each specimen undergoes a rapid elastic‐plastic transition, while the reduction in initial displacement reflects anelastic behavior. Notably, as illustrated in Figure 2b, the relationship between creep displacement (h) and holding time (t) reveals that during the load‐holding period, the creep displacement of the as‐fabricated (room‐temperature) micropillar is 48 nm. Upon heat treatment at 100°C, the creep displacement increases to 68 nm, representing a 42% rise relative to the room‐temperature state. At 200°C, the displacement further rises to 101 nm, marking a 110% increase. However, when the heat treatment temperature is elevated to 300°C, the creep displacement does not continue to increase but instead decreases to 42 nm, a 13% reduction compared to the room‐temperature value. In summary, under the given heat treatment conditions, the creep displacement of copper micropillars initially increases and then decreases with rising temperature. Based on this trend, it can be inferred that a heat treatment temperature of 300°C is sufficient to reduce the creep displacement of copper micropillars.

In nanoindentation creep tests, the displacement‐time (h–t) curve exhibits two distinct stages: first, a transient creep stage where displacement increases rapidly, followed by a steady‐state creep stage in which displacement increases approximately linearly with time, as is shown in Figure 2b. The creep behavior observed in the current study is highly consistent with previous nanoindentation creep investigations conducted at room temperature on bulk metals (such as Ag‐, Ni‐, Zn‐, and Al‐based alloys) [31, 32, 33, 34, 35], as well as on various amorphous materials (including Zr‐, Pd‐, and rare earth (RE)‐based bulk metallic glasses) [36, 37]. It is noteworthy that the nanoindentation creep process differs from conventional tensile creep, which typically includes three stages: in addition to the transient and steady‐state creep stages similar to those in nanoindentation, there is also a tertiary creep stage characterized by a significant increase in creep rate over time, ultimately leading to specimen fracture. In the present study, however, the nanoindentation creep tests do not involve fracture phenomena [38].

This concept is based on the assumptions of the expanding cavity model and has been further developed and refined by Charitidis and subsequent researchers [39, 40]. Under these assumptions, the steady‐state creep behavior conforms to the classical power‐law creep model. In the steady‐state creep stage, the strain rate remains essentially constant, resulting in a nearly linear creep curve. This stability clarifies the relationship between strain rate and stress, enabling a more accurate reflection of the material's creep mechanism. The applicability of the power‐law creep equation is strongest in this stage, and the stress exponent (n) can be derived from the power‐law creep equation [41, 42, 43].

(1) $$\frac{d\varepsilon \left(h_{p}\right)}{dt}=A\cdot \sigma^{n}$$

Taking the logarithm of both sides of the equation, the above equation can be equivalently transformed as follows:

(2) $$\ln\left(\frac{d\varepsilon \left(h_{p}\right)}{\mathrm{dt}}\right)=\ln A+n\cdot \ln\sigma$$

In the above equation, $\frac{d\varepsilon (h_{p})}{dt}$ represents the steady‐state creep strain rate, which in nanoindentation testing can be expressed by the following differential equation:

(3) $$\frac{d\varepsilon \left(h_{p}\right)}{dt}=\frac{dh_{p}/h_{p}}{dt}=\frac{1}{h_{p}}\;\cdot \frac{dh_{p}}{dt}$$

For brevity, in this study, the steady‐state creep strain rate is simplified as:

(4) $$\dot{\varepsilon }=\frac{1}{h_{p}}\cdot \frac{dh_{p}}{dt}$$

h_p_ is the contact depth of the indenter, which can be calculated from the following equation:

(5) $$h_{p}=h_{t}\;-\frac{\gamma \cdot P}{S}$$

In the above equation, h_t_ is the total displacement in nanoindentation, γ is a correction factor accounting for the tip effect of the indenter, and S is the contact stiffness derived from the unloading curve. In this work, γ is set to 0.72 for the Berkovich indenter.

σ represents the instantaneous stress generated during indentation with a Berkovich indenter, which is equivalent to the nanoindentation hardness (H). It can be calculated using the following formula:

(6) $$\sigma =H=\frac{P}{C\cdot h_{p}^{2}}\;$$

In the above equation, P is the applied load during the test (i.e., 80 mN); C is the indenter tip area coefficient, which was calibrated through testing on standard fused silica. In the current experimental setup, C is equal to 24.5.

**FIGURE 3:**
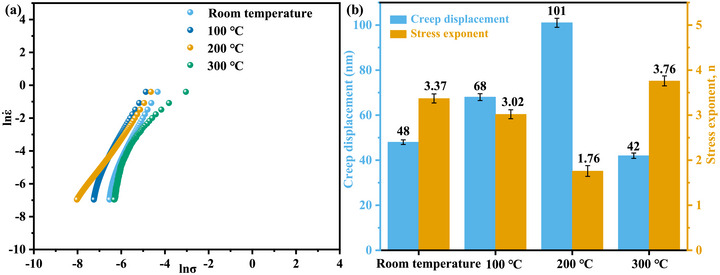
The derived ln($\dot{\varepsilon }$)‐ln(σ) curves (a) and histogram of stress exponent (n) and creep displacement of copper micropillars before and after heat treatment.

As can be seen from Equation (2), the stress exponent (n) can be calculated from the slope of the ($\ln(\dot{\varepsilon })-\ln\sigma$) curve. According to Figure 2, during the steady‐state stage, the strain rate remains relatively stable, making the experimental data easier to collect and process, and the requirements for measurement accuracy are comparatively lower. Based on this, in the present study, the (n) value for each specimen was determined from the latter half of the steady‐state creep segment. As shown in Figure 3b, as the heat treatment temperature increases to 200°C, the stress exponent decreases from 3.37 to 1.76. When the heat treatment temperature is further increased to 300°C, the stress exponent rises to 3.76. The physical significance of the stress exponent lies in its ability to indicate the creep mechanism: diffusion creep typically corresponds to an (n) value of approximately 1, grain boundary sliding creep to an (n) value of about 2, and dislocation creep to an (n) value generally greater than 3 [44, 45, 46]. This implies that after heat treatment at 200°C, the creep behavior of the copper micropillars undergoes a mechanistic transition, shifting from dislocation creep to grain boundary sliding creep.

Generally, in traditional metallic crystalline materials, a higher value of the stress exponent (n) indicates greater creep resistance. As shown in the nanoindentation creep test results in Figure 3b, a significant influence of heat treatment temperature on the material's creep behavior is observed: as the heat treatment temperature increases to 200°C, the creep displacement exhibits a monotonic increasing trend (from 48 to 101 nm), yet drops to 42 nm at 300°C. The non‐monotonic variation in creep displacement, combined with the evolution of the stress exponent (n), collectively suggests that the dominant creep mechanism of the material may undergo a transition with temperature. Specifically, at a heat treatment temperature of 200°C, the lower (n) value (1.76) implies enhanced grain boundary diffusion or viscous sliding mechanisms, whereas at 300°C, the increased (n) value (3.76) likely reflects the re‐emergence of dislocation activity as the dominant mechanism. To further elucidate this temperature‐dependent microscopic mechanism, the next step involves calculating the activation volume to quantitatively analyze the effective volume change of deformation carriers during the thermally activated process, thereby establishing a structure‐property relationship linking heat treatment temperature, deformation mechanism, and creep performance.

The determination of thermodynamic activation parameters is typically based on three experimental methods: stress relaxation tests, creep tests, and strain rate jump tests. It must be emphasized that during relevant differential operations, the internal microstructure of the material—particularly the dislocation density—must remain in a stable state. Experimental studies have shown that when testing protocols that alter the microstructure or dislocation density are employed (e.g., comparing data from creep tests conducted at different strain rates), the resulting results often contain systematic errors [47]. It is worth noting that although transient testing methods also induce changes in dislocation configurations during the determination of activation parameters (such changes are manifested as transient responses during strain rate switching), these methods have been extensively validated in practical applications [48]. Similarly, while stress relaxation tests are accompanied by a continuous reduction in dislocation density, their results still hold a reliable reference value. In the nanoindentation testing process, the shear stress can serve as a transfer parameter to calculate the activation volume of the shear transformation zone (STZ). The activation volume (V*) of the STZ can be expressed as [49]:

(7) $$V^{*}=M\frac{k_{B}T}{m\cdot \sigma_{f}}$$

In this context, K_B_ is the Boltzmann constant, and T is the absolute temperature in Kelvin (298.15 K was used in the experiment). In nanoindentation testing, the von Mises yield criterion is applied (instead of the Taylor relation commonly used in uniaxial tests), giving a value of $M=\sqrt{3}$. The flow stress σ_*f*_ can be calculated from the nanoindentation hardness using the Tabor relation [50], expressed as: 𝐻 = 3σ_*f*_. The strain rate sensitivity m is the reciprocal of the stress exponent n.

**FIGURE 4:**
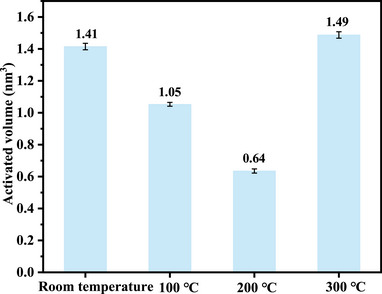
The histogram of the activation volume for copper micropillars under different heat treatment temperatures.

Figure 4 presents a histogram of the activation volume for copper micropillars under different heat treatment temperatures. The activation volume initially decreases from 1.41 nm^3^ in the as‐received state to 0.64 nm^3^ after treatment at 200°C, representing a reduction of 55% compared to the untreated condition. Subsequently, it increases to 1.49 nm^3^ at 300°C. This non‐monotonic variation is strongly correlated with the evolution of creep displacement (48 nm→101 nm→42 nm) and stress exponent (3.37→1.76→3.76). When the heat treatment temperature is raised to 200°C, the marked reduction in activation volume (0.64 nm^3^) and the lower stress exponent (1.76) suggest that the deformation is achieved through extremely short‐range, rapid atomic diffusion along grain boundaries or the emission and movement of dislocations at grain boundary steps. These events occur on the scale of a few atomic spacings, involve very few atoms, and thus the associated activation volume is naturally very small. The material no longer relies primarily on intragranular dislocations struggling to overcome obstacles; instead, it accommodates deformation through the “relative flow” of grain boundaries, resulting in greater deformation under the same conditions. In contrast, the anomalous increase in activation volume at 300°C (1.49 nm^3^) may be attributed to the dominant mechanism being dislocation climb, which involves long‐range diffusion and the overcoming of larger obstacles (such as subgrain boundaries), leading to a larger activation volume. Notably, this structural evolution differs fundamentally from the activation volume variation mechanism observed in metallic glasses, where the activation volume is primarily governed by the redistribution of free volume. In pure copper, however, the changes in activation volume are mainly attributed to the interactions between dislocations and grain/subgrain boundaries. This result indicates that although the magnitude of activation volume variation in copper (∼1.5 nm^3^) is significantly larger than that in metallic glasses (∼0.03 nm^3^), it is still fundamentally dominated by the dynamic behavior of defects intrinsic to crystalline materials, such as dislocations and grain boundaries [51, 52, 53].

To place the creep performance of the copper micropillars in this study within the context of existing research and to objectively evaluate their relative level, we have compiled the creep‐related metrics of representative copper materials under various fabrication routes for benchmarking comparison, as shown in Table 1. It should be emphasized that the conditions for obtaining the stress exponent in the literature vary significantly: on the one hand, some data are derived from high‐temperature creep tests, with temperature ranges reaching up to 473–923 K or even higher, as shown in **Group I** of Table 1; on the other hand, the data in this study are from creep measurements taken at room temperature, as shown in **Group II** of Table 1. Given that temperature and testing methods significantly influence the physical meaning of creep mechanisms and power‐law parameters, the high‐temperature creep data in **Group I** of Table 1 are primarily used here for background reference comparison, rather than for direct ranking comparison of the stress exponent with our results. Under directly comparable room temperature/near‐room temperature conditions, the stress exponents of pure copper or electroplated copper composite systems in the literature typically fall within the range of 2–3 or higher (see **Group II** in Table 1), reflecting the mechanism differences under various processing and microstructural states. Although the stress exponent obtained for “Copper Coatings Reinforced with Pigment Particles” is superior to that of this study, this process cannot be used for through‐hole electroplating to fabricate copper micropillars. The copper pillars fabricated by laser additive manufacturing have low precision, high roughness, and cannot be miniaturized. In contrast, the high‐aspect‐ratio pure copper micropillars (with a diameter of approximately 50 µm and a height of approximately 300 µm) fabricated by the TGV process in this study achieved a stress exponent of 3.76 after heat treatment at 300°C, indicating that under strict microscale geometric and integration constraints, this study can still achieve an engineering‐relevant enhancement in the anti‐creep response. Meanwhile, from a structural scale perspective, most of the comparative materials in the literature are in macroscopic forms such as bulk, rod, or foil, and their processing routes make it difficult to achieve pin fin miniaturization. Therefore, the results in Table 1 further demonstrate that this study has achieved a good balance among the anti‐creep performance, geometric miniaturization, and compatibility with the TGV microfabrication process for copper micropillars.

**TABLE 1:** A comparison of the stress exponents of pure copper and copper alloys produced via various fabrication techniques.

Ref.	Fabrication process	Stress exponent	Temperature (K)	Material composition	Shape	Group	Material dimensions (mm)
[24]	Hot rolling and cold rolling	6–8	725–855	Cu–0.3Cr–0.1Ag Alloy	sheet	Group I	With a thickness of 5
[54]	hot forged	5.2–26.6	473–923	Copper‐chromium alloy	rod	Group I	70 × Φ 3
[55]	wire‐drawn	8.11	773	Cu‐20%Nb alloys	rod	Group I	With a diameter of 13
[25]	Cold rolling	2–3	293–323	Pure copper	foil	Group II	15 × 3 × 0.075
[56]	Cold drawing	2.39	298	Pure copper	rod	Group II	100 × Φ3.6
[57]	Electrodeposition	9.41	298	Copper Coatings Reinforced with Pigment Particles	foil	Group II	Not reported
[58]	Laser powder bed additive manufacturing	10–50	298	copper‐carbon nanotube composite	cube	Group II	10 ×10 × 10
This study	TGV	3.76	298	Pure copper	Micro‐pillar	Group II	0.3 × Φ 0.05

### 2.3 Viscoelastic Behavior Analysis

The analysis of activation volume reveals the essence of the thermal activation mechanism during plastic deformation, providing a crucial perspective for understanding the material's microscopic deformation behavior. However, this analysis primarily focuses on deformation characteristics under steady‐state or quasi‐static conditions, making it insufficient to fully characterize the time‐dependent behavior of the material under sustained loading. To further investigate the viscoelastic response of the material during nanoindentation creep, the experimentally obtained indentation depth‐time (h‐t) curves can be theoretically analyzed using a series‐connected generalized Kelvin model. This mechanical model consists of several Kelvin‐Voigt units connected in series, each of which is composed of an elastic spring and a viscous dashpot arranged in parallel [45].

In this study, a classical modeling approach incorporating two series‐connected Kelvin units is adopted for the creep analysis of copper micropillars. Through the constitutive relationship established by this generalized Kelvin model, the h‐t curves obtained from nanoindentation creep tests can be quantitatively expressed as the following mathematical model [59]. Based on this model, a mathematical relationship between creep displacement (h) and time (t) is established, laying a theoretical foundation for the subsequent quantitative analysis of the material's creep compliance and hysteresis spectrum.

(8) $$h\;\left(t\right)=h_{0}+\sum_{i=1}^{n}h_{i}\left(1-e^{-\frac{t}{\delta_{i}}}\right)+\frac{t}{\mu_{0}}$$

**FIGURE 5:**
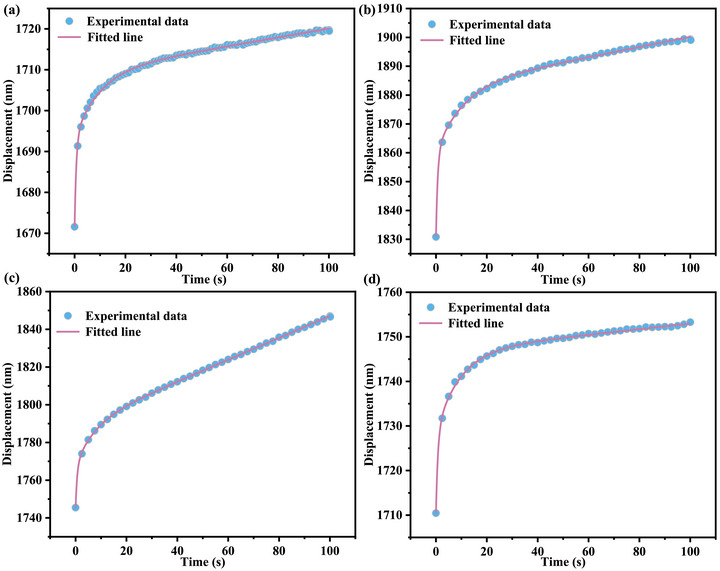
Experimental and fitted creep displacement‐holding time curve: (a) Room temperature; (b) 100°C; (c) 200°C; (d) 300°C.

According to the mathematical formulation of the generalized Kelvin model, each parameter in the aforementioned equation has a clear physical meaning: h_0_ represents the instantaneous indentation depth corresponding to the initial spring element; h_i_ and δ_i_ describe the creep displacement characteristics and time‐delay effect of the (i)‐th Kelvin unit, respectively (in this model, a two‐unit series configuration is adopted, hence (n = 2)); and µ_0_ reflects the intrinsic rheological behavior of the terminal viscous damping element. As shown in Figure 5, the experimentally measured (h)–(t) curve is in high agreement with the fitted results of the model, with specific parameters detailed in Table 2. To further support the interpretation of the rheological behavior transition between 200°C and 300  °C, additional nanoindentation creep tests were conducted at an intermediate temperature of 250  °C, with the results provided in the Supporting Information (Figures S1 and S2 and Table S1). It should be noted that the parameter µ_0_ exhibits a distinctly non‐monotonic evolution with increasing annealing temperature: it continuously decreases from 9.26 at room temperature to a minimum value of 1.73 at 200  °C, and then significantly increases to 16.08 when the annealing temperature is raised further to 300  °C. This trend, from a rheological perspective, indicates that the copper micropillars become more susceptible to viscous flow and exhibit reduced creep resistance upon annealing from room temperature up to 200  °C. In contrast, after annealing at 300  °C, the system develops substantially higher viscous resistance, leading to enhanced creep resistance.

**TABLE 2:** The fitting results of parameters in Equation (8).

	h_0_	h_1_	δ_1_	h_2_	δ_2_	µ_0_	R^2^
Room temperature	1671.57	15.92	9.72	21.78	0.68	9.26	0.99
100°C	1830.83	22.56	10.60	29.41	0.69	5.76	0.99
200°C	1745.46	21.46	7.35	22.16	0.62	1.73	0.99
300°C	1710.44	18.07	9.47	18.24	0.79	16.08	0.99

After obtaining the functional relationship between creep displacement and time, the creep compliance, C(t), as a key parameter characterizing the viscoelastic constitutive behavior of the material, can be further calculated using a specific formula. Creep compliance reflects the evolution of strain with time under constant stress and serves as an important indicator for evaluating the material's resistance to creep deformation. In this study, based on the displacement–time relationship derived from the Kelvin model and combined with the applied load parameters, the creep compliance as a function of time was calculated. This process enables the quantitative characterization of the material's macroscopic viscoelastic behavior and provides essential constitutive data support for subsequent hysteresis spectrum analysis.

According to reference [60], the mathematical expression for the creep compliance is defined as:

(9) $$C\;\left(t\right)=\frac{A_{0}}{P_{0}h_{in}}\;h\left(t\right)$$

Substituting Equation (8) into the above expression yields:

(10) $$\begin{array}{c} C\left(t\right)=\frac{A_{0}}{P_{0}h_{in}}\;\left(h_{0}+h_{1}\left(1-e^{-t/\delta_{1}}\right)+h_{2}\left(1-e^{-t/\delta_{2}}\right)+t/\mu_{0}\right) \end{array}$$

In the above equation, A_0_ is the contact area (determined by curve fitting, A_0_ = 24.5*h(*t*)^2^). P_0_ is the load applied during the holding stage (80 mN), and h_in_ denotes the indenter depth before entering the holding stage.

**FIGURE 6:**
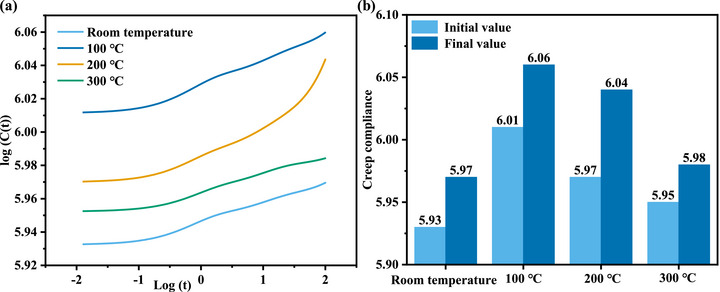
Compliance spectra (a) and initial and final values of creep compliance (b) under different heat treatment temperatures.

Figure 6a shows the variation of creep compliance with time. It can be observed that in the initial stage (from 10^−^^2^ s to 10^−^^1^^.^^5^ s), the creep compliance changes gently and remains nearly constant for a period. This indicates that although the heat treatment temperatures differ, the mechanical behavior of the copper micropillars is predominantly governed by instantaneous elasticity. At this stage, reversible elastic distortion of the atomic lattice bonds occurs, while thermally activated motion of defects such as dislocations and grain boundaries has not yet been initiated. However, as the holding time increases, the curves for all samples show that creep compliance increases with time, though the magnitude of increase and the initial values vary. As illustrated in Figure 6b, the initial and final creep compliance values for each copper micropillar sample are presented. Through calculation, we find that the increments in creep compliance during the creep process differ with heat treatment temperature. At room temperature, this increment is only 0.04. After heat treatment at 100°C, the increment rises to 0.05. Following heat treatment at 200°C, the creep compliance increment reaches a peak value of 0.07. However, as the heat treatment temperature further increases to 300°C, the increment drops to 0.03. As is well known, creep compliance reflects the accumulated deformation of a material over time under constant stress. This suggests that after heat treatment at 300°C, the micro copper pillar exhibits the slowest deformation rate per unit time during the creep process.

Although creep compliance can effectively describe the viscoelastic response of a material in the time domain, it is difficult to directly reveal the distribution characteristics of different microscopic relaxation processes within the material. To further analyze the energy dissipation mechanism in the frequency domain and elucidate the viscoelastic response characteristics under dynamic loading, this study transforms the creep compliance into a retardation spectrum for analysis. By applying Fourier or Laplace transforms to the creep compliance, the retardation spectrum maps time domain information into the frequency domain, thereby revealing energy dissipation peaks corresponding to different relaxation times within the material. This analysis not only helps identify dominant viscoelastic mechanisms in the material, such as grain boundary sliding and dislocation motion, but also provides an important basis for evaluating the effects of heat treatment processes on the material's microstructure and macroscopic mechanical properties.

The retardation spectrum of the micro‐copper pillar can be derived from the following compliance equation [38].

(11) $$P\left(\tau \right)=dC\left(t\right)/d\ln t-d^{2}C\left(t\right)/d{\left(\ln t\right)}^{2}{|}_{t=2\tau }$$

The above equation can be rewritten as:

(12) $$\begin{array}{c} P\left(t\right)=\left[\left(1+\frac{t}{\tau_{1}}\right)\frac{h_{1}}{\tau_{1}}e^{-t/\tau_{1}}+\left(1+\frac{t}{\tau_{2}}\right)\frac{h_{2}}{\tau_{2}}e^{-t/\tau_{2}}\right]\;\frac{A_{0}}{P_{0}h_{in}}t{|}_{t=2\tau } \end{array}$$

**FIGURE 7:**
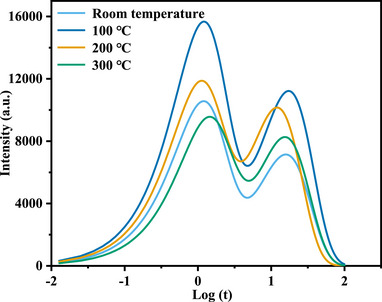
Retardation spectra under different heat treatment temperatures.

Figure 7 presents the hysteresis spectra of the specimens. Two distinct energy dissipation peaks are observed in the spectra, located in the short‐time scale (Log(t) ≈ 0, approximately 1 s) and long‐time scale (Log(t) ≈ 1.5, approximately 30 s) regions, respectively. These peaks reflect two dominant microscopic relaxation mechanisms within the material. On the one hand, a rightward shift in the relaxation time of the spectral peaks can be observed across all specimens. This indicates that the microstructural relaxation processes associated with the peaks become slower. After heat treatment at 300°C, the relaxation rate of the micro‐copper column specimen decreases, requiring a longer time to reach the peak energy dissipation response. This suggests that more microstructural barriers may impede dislocation motion within the material. On the other hand, the intensity of the spectral peaks typically reflects the material's internal energy dissipation capability under specific conditions. A higher peak intensity signifies greater energy dissipation, indicating that more inelastic mechanisms are active within the material, with defects such as grain boundaries and dislocations being more mobile, thereby facilitating plastic deformation. According to the calculated hysteresis spectral peak intensities, the peak intensities of the specimens after heat treatment at 100°C and 200°C are significantly higher than those after treatment at 300°C. Combined with the creep compliance results, it is evident that the specimen treated at 300°C exhibits lower energy dissipation and slower deformation rates under mechanical excitation, resulting in smaller creep displacement and displaying dislocation climb‐type creep behavior under constant load.

Interestingly, however, the peak intensity in the retardation spectrum of the sample after heat treatment at 100°C, as shown in Figure 7, is higher than that of the sample treated at 200°C. This result appears to contradict the corresponding creep displacement data. Specifically, for pure copper, the intensity of the creep retardation spectrum after 100°C heat treatment exceeds that of the 200°C treated sample, indicating that the former undergoes a more pronounced energy dissipation process at the lower treatment temperature. Nevertheless, despite this stronger energy dissipation, the 100°C treated sample exhibits lower creep displacement than the 200°C treated one during the creep process. This suggests that the relationship between energy dissipation and macroscopic plastic deformation is not simply positively correlated; even with higher energy dissipation, significant plastic deformation may not necessarily occur. Therefore, to gain deeper insight into this contradictory phenomenon, it is essential to further analyze the microstructural changes in the material under the two heat treatment conditions, such as lattice mismatch, grain boundary distribution, and grain boundary characteristics, in order to reveal their regulatory effects on creep behavior.

### 2.4 Microstructure Analysis

As described in Section 2.3, the heat treatment process significantly affects the creep properties of copper micropillars, resulting in a trend where their creep resistance first decreases and then increases. However, the evolution of macroscopic properties is inevitably rooted in profound changes in the microstructure. To deeply reveal the underlying micromechanisms responsible for this performance improvement, it is necessary to systematically characterize the microstructure. Therefore, this study further employs electron backscatter diffraction (EBSD) and transmission electron microscopy (TEM) to analyze the microstructure of the samples before and after heat treatment. The aim is to elucidate the evolution patterns of the microstructure within the copper micropillars induced by heat treatment and to establish the intrinsic correlation between these microstructural features and the macroscopic creep behavior.

**FIGURE 8:**
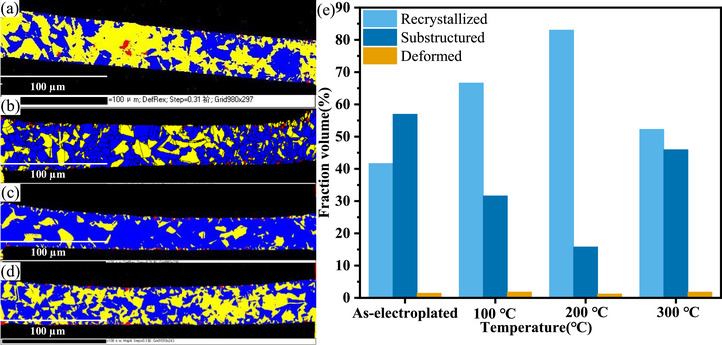
EBSD results of the copper micropillars cross‐section at room temperature, 100°C, 200°C, and 300°C: (a–d) recrystallization maps, (e) summarised results of recrystallized, substructured, and deformed region.

Figure 8a–d show EBSD images of the recrystallized, substructured, and deformed regions in copper micropillars before and after heat treatment. In the images, blue represents the recrystallized regions, yellow the substructured regions, and red the deformed regions. Figure 8e presents a histogram comparing the area fractions of recrystallized, substructured, and deformed regions in the micropillars before and after heat treatment. After heat treatment at 100°C, the fraction of recrystallized regions increased to 66.6%. When the temperature was raised to 200°C, the recrystallized fraction further increased to 83%. Interestingly, after heat treatment at 300°C, the recrystallized fraction did not continue to rise but instead decreased to 52.3%. Meanwhile, the fraction of substructured regions initially decreased to 15.8% after heat treatment at 200°C, but after treatment at 300°C, it did not continue to decrease; instead, it rose to 46%. In summary, during the heat treatment process, the fraction of recrystallized regions in the copper micropillars exhibits a trend of first increasing and then decreasing, while the fraction of substructured regions shows the opposite trend—first decreasing and then increasing.

During the heat treatment process, the evolution of recrystallized and substructured regions in the samples exhibits a clear temperature dependence. After heat treatment at 200°C, the fraction of recrystallized regions reaches its maximum, while the proportion of substructured regions decreases significantly. This phenomenon corresponds to the increase in creep displacement, indicating that the recrystallization process helps release dislocation stress and promote dislocation slip, thereby enhancing the material's plastic deformation capability [61]. However, when the heat treatment temperature is further increased to 300°C, the recrystallized fraction decreases instead, while the substructured regions increase again, which may be related to the re‐proliferation of dislocations. Therefore, the effect of heat treatment on creep performance cannot be explained solely by the recrystallization mechanism; rather, it is closely related to the interplay of multiple factors, including substructure evolution, dislocation density, and grain boundary characteristics. To further elucidate the regulatory roles of these microstructural mechanisms on creep behavior, it is necessary to systematically analyze low‐angle grain boundaries (LAGBs) and high‐angle grain boundaries (HAGBs), in order to clarify the functions of different grain boundary types in dislocation motion and plastic deformation. In other words, by examining the evolution of grain boundaries, we can identify the underlying causes of changes in the creep behavior of copper micropillars.

**FIGURE 9:**
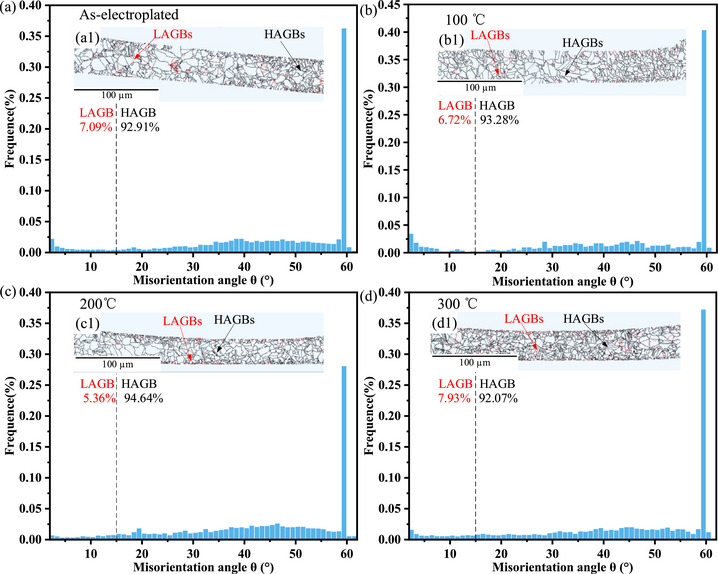
EBSD results of the copper micropillars cross‐section at room temperature, 100°C, 200°C, and 300°C: (a–d) grain misorientation angle distribution histograms, (a1,b1,c1,d1) grain boundary maps.

As shown in the grain boundary maps in Figure 9a–d, black lines represent high‐angle grain boundaries (HAGBs), defined as those with a misorientation angle θ greater than 15°, while red lines represent low‐angle grain boundaries (LAGBs), defined as those with θ in the range of [2°–15°]. When the heat treatment temperature is 100°C, the fraction of LAGBs decreases to 6.72%. Upon heat treatment at 200°C, the LAGB fraction continues to decrease to 5.36%, and the proportion of substructured regions further declines. That is, as the heat treatment temperature increases, the mobility of grain boundaries is enhanced, and the recrystallized fraction rises, which consumes the substructures closely associated with LAGBs, leading to a continued reduction in both LAGB and substructure fractions. However, it should be emphasized that when the temperature is raised to 300°C, the recrystallized fraction begins to decline, while the proportion of LAGBs starts to rise. Since LAGBs are typically formed by the arrangement of dislocations within the crystal, their movement and accumulation contribute to the formation of such boundaries [62]. Therefore, the variation in dislocation density can be understood through an analysis of the geometrically necessary dislocations (GND) within the copper micropillars.

**FIGURE 10:**
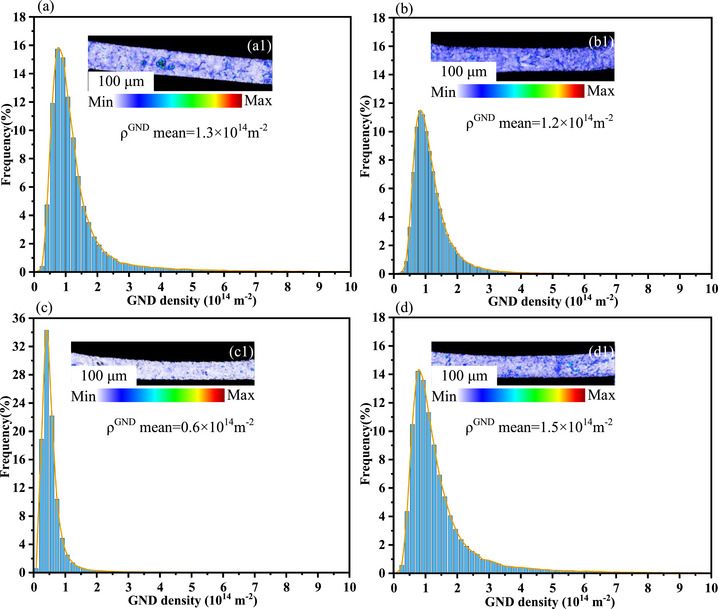
EBSD results of the copper micropillars cross‐section at room temperature, 100°C, 200°C, and 300°C: (a–d) calculated mean GND (geometrically necessary dislocations) density distribution histograms, (a1,b1,c1,d1) GND maps.

Figure 10 shows the geometrically necessary dislocation (GND) maps of the cross‐sections of copper micropillars before and after heat treatment. It can be seen that, with increasing heat treatment temperature, the average GND density exhibits a trend similar to the fraction of substructured regions shown in Figure 8. In the temperature range from room temperature to 200 °C, the average GND density decreases from 1.3 × 10^14^ *m* ^−2^ to 0.6 × 10^14^ *m* ^−2^, indicating an overall reduction in dislocation density. This is likely attributed to enhanced dislocation mobility at elevated temperatures in this regime, enabling small‐angle grain boundaries to absorb dislocations and transform into high‐angle grain boundaries, thereby reducing both the overall strain and dislocation density. Interestingly, when the heat treatment temperature is further increased to 300 °C, thermal strain induced by the coefficient of thermal expansion mismatch between glass and copper intensifies. As a result, the generation of new dislocations exceeds the rate at which they are consumed by transformation into high‐angle grain boundaries, leading to dislocation accumulation and an increase in the average GND density to 1.5 × 10^14^ *m* ^−2^.

To gain deeper insight into the intrinsic relationship between microstructural evolution and creep behavior in copper micropillars—and to enable quantitative mechanistic analysis—we employ the classical power‐law creep theory [63, 64], which provides a robust physical foundation for describing dislocation‐motion‐dominated creep behavior. The steady‐state creep strain rate, ${\dot{\varepsilon }}_{c}$, is expressed as follows:

(13) $${\dot{\varepsilon }}_{c}=B\cdot \exp\left(\begin{array}{c} -\frac{Q}{\mathrm{RT}} \end{array}\right)\cdot {\left(\frac{\sigma }{\hat{\sigma }}\right)}^{n}$$

(14) $$\hat{\sigma }=M_{t}\alpha Gb\sqrt{\rho }$$

In the above equation, n is the creep stress exponent, $\hat{\sigma }\;$ is a function of dislocation density, and *M_t_* is the Taylor factor. In this study, based on EBSD measurements, *M_t_* takes the values of 3.12, 3.12, 3.13, and 3.14 with increasing heat treatment temperature, respectively. Both B and α are numerical constants, Q is the activation energy for creep, R is the universal gas constant, G is the shear modulus, and ρ denotes the dislocation density. It follows that:

(15) $${\dot{\varepsilon }}_{c}\propto {M_{t}}^{-n}\times \rho^{-n/2}$$

**FIGURE 11:**
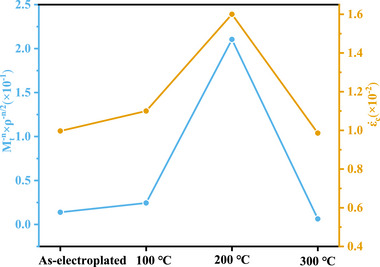
The variation of the creep strain rate (${\dot{\varepsilon }}_{c}$) and *M_t_* ^−n^*ρ^−*n*/2^ with different heat treatment temperatures.

In crystal plasticity theory, the total dislocation density (ρ) is typically decomposed into two components: the geometrically necessary dislocation (GND) density and the statistically stored dislocation (SSD) density. GNDs arise to accommodate orientation gradients within grains, whereas SSDs are randomly stored dislocations generated during plastic deformation. At the micropillar scale investigated in this study, deformation is typically accompanied by pronounced grain boundary incompatibility and strong orientation gradients; consequently, GNDs dominate the total dislocation density and play a pivotal role in determining material strength and deformation behavior. Based on this consideration, we adopt the GND density measured via electron backscatter diffraction (EBSD) as a proxy for the total dislocation density (ρ). This approach enables a quantitative analysis of how microstructural evolution influences creep resistance. Figure 11 presents the variation of the creep strain rate and the *M_t_* ^−*n*^ × ρ^−*n*/2^ with different heat treatment temperatures. After heat treatment from room temperature up to 200°C, the value of *M_t_* ^−*n*^ × ρ^−*n*/2^ exhibits an increasing trend. In contrast, after heat treatment at 300 °C, this value decreases. According to Equation (15), the creep strain rate should therefore first increase and then decrease during the heat treatment process—a trend that aligns well with our calculated results for the creep strain rate.

**FIGURE 12:**
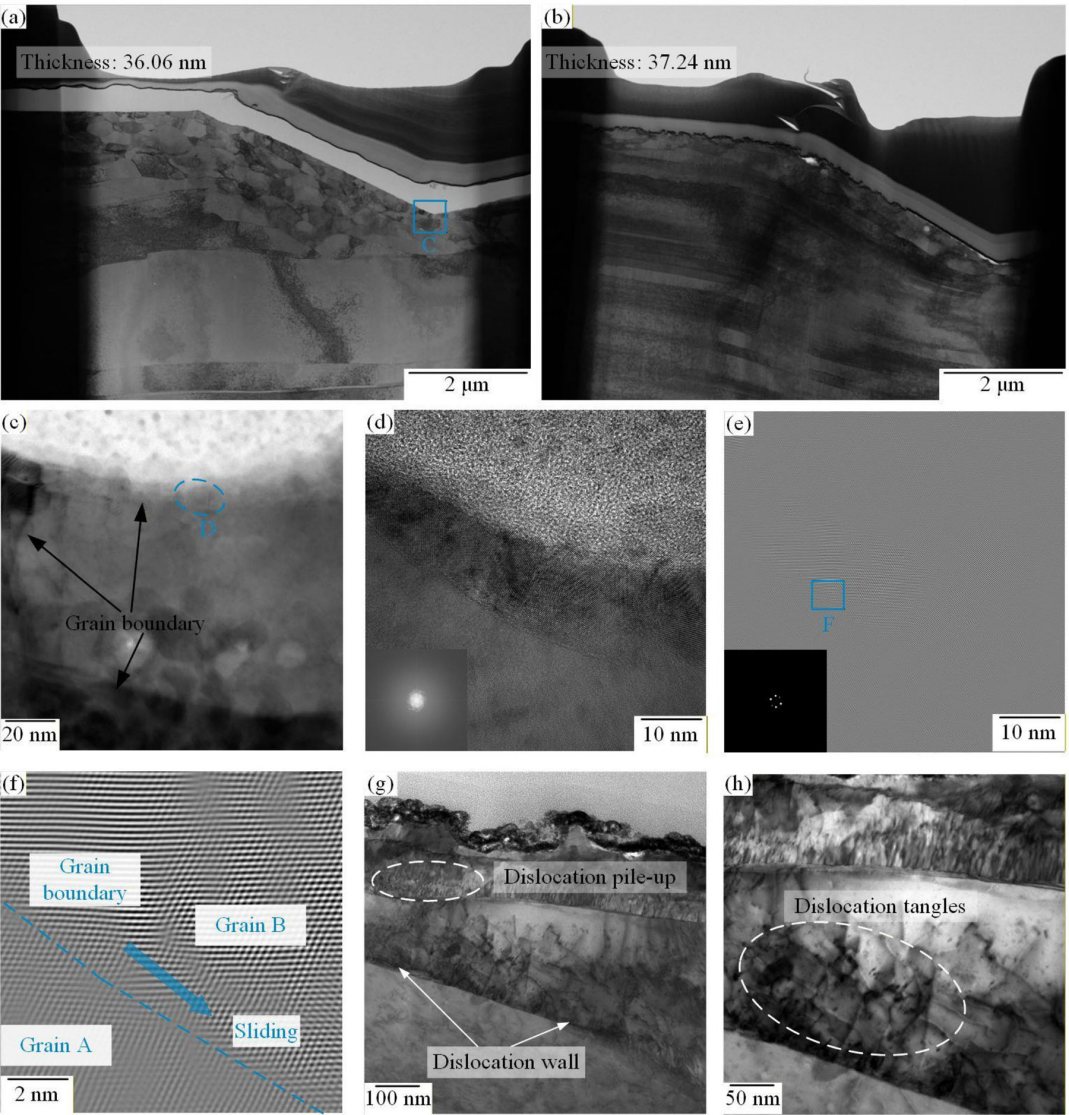
Cross‐sectional TEM foil of the nanoindentation‐tested copper micropillar (a) 200°C and (b) 300°C, (c) TEM image of area C, (d) original HRTEM micrograph obtained from area D, (e) corresponding IFFT image, (f) area F in the IFFT image, (g,h) TEM images of the dislocation structure of the copper micro‐pillars after heat treatment at 300°C.

To gain deeper insights into the evolution of microstructural differences in copper micropillars during the transition of creep mechanisms, we performed transmission electron microscopy (TEM) characterization on the indent regions of micropillar samples subjected to different heat treatment temperatures. To ensure the accuracy of the analysis, the TEM foil was prepared using a focused ion beam (FIB) system and the lift‐out technique, as shown in Figure 12a,b. The TEM foils were thinned to uniform thicknesses of 36.06 and 37.24 nm, respectively, thus eliminating contrast variations due to mass‐thickness effects in TEM images [65]. By comparing the TEM images, it can be observed that the dislocation density after heat treatment at 300°C is significantly higher than that after heat treatment at 200°C. Figure 12d,e show the high‐resolution transmission electron microscopy (HRTEM) images and corresponding fast Fourier transform (FFT) patterns of region D in Figure 12c. The original HRTEM images were filtered using mask functions in Digital Micrograph software, followed by inverse fast Fourier transform (IFFT), as shown in Figure 12f. Local atomic displacements across grain boundaries can be observed, manifesting as relative shifts in atomic planes between adjacent grains. These displacements are confined to the grain boundary regions and do not uniformly extend into the grain interiors, indicating an inelastic and non‐uniform lattice distortion. Such localized atomic rearrangements at grain boundaries are widely recognized as characteristic features of grain boundary‐mediated deformation mechanisms. Combined with the TEM observation results shown in Figure 12c (corresponding to region C in Figure 12a, where intragranular dislocation activity is extremely limited, these findings suggest that grain boundary processes play a critical role in the creep deformation of copper micropillars after heat treatment at 200°C. However, following heat treatment at 300°C, more pronounced dislocation networks appear within the grains, accompanied by dislocation pile‐ups and entanglements at the grain boundaries (Figure 12g,h). These dislocation‐grain boundary interactions exert pinning effects on grain boundary migration or sliding, thereby altering the deformation accommodation mode and providing direct mechanistic evidence for dislocation‐dominated creep mechanisms.

In summary, integrating the analyses from Sections 2.2–2.4, it is evident that different heat treatment temperatures significantly influence the microstructural evolution within copper micropillars, including dynamic changes in substructures, LAGBs, and GND, which in turn dictate their viscoelastic behavior and creep mechanisms. At a heat treatment temperature of 100°C, dislocations begin to move and annihilate, yet a relatively high dislocation density remains within the copper micropillars. During this stage, dislocations transform into mobile dislocations, thereby enhancing energy dissipation capacity at short timescales. Additionally, the reduction in low‐angle grain boundaries may release some internal stress, further promoting dislocation slip activity. Consequently, dislocation slip during the holding stage becomes the dominant mechanism of energy dissipation, resulting in a higher intensity of the retardation spectrum. As the temperature increases to 200°C, the proportions of LAGBs, substructures, and GND decrease, while the fraction of static recrystallization increases. This process facilitates dislocation climb and annihilation, reducing dislocation density, while simultaneously expanding the statically recrystallized regions and allowing more complete relaxation of grain boundaries. As a result, the contribution of grain boundary sliding to creep is enhanced, leading to increased creep displacement, which aligns with the grain boundary sliding creep mechanism revealed by stress exponent calculations.

However, when the heat treatment temperature is further increased to 300°C, the increase in dislocation density exceeds its consumption by recrystallization, leading to a reduction in the recrystallized fraction and a consequent rise in dislocation density. A large number of dislocations accumulate near grain boundaries, forming dislocation pile‐ups, which exert a pinning effect on the grain boundaries and impede grain boundary sliding. As a result, the creep mechanism transitions to a dislocation‐type creep, accompanied by a significant reduction in creep displacement. This transition is highly consistent with the variation in the two energy dissipation peaks observed in the retardation spectra: heat treatment at 100°C and 200°C promotes the recovery process, releases internal stresses, and activates both short‐term and long‐term relaxation activities of dislocations and grain boundaries; in contrast, heat treatment at 300°C introduces new dislocations and substructures, suppressing relaxation activity and indicating that the material's microstructure tends toward stabilization with a diminished viscoelastic response. These findings not only reveal the underlying mechanisms by which heat treatment temperature affects the viscoelastic properties of the material but also provide an important basis for optimizing heat treatment processes to tailor the microstructure and mechanical properties.

### 2.5 Thermal Conductivity Analysis

Through the characterization of the microstructure of heat‐treated copper micropillars, the underlying micromechanisms governing the evolution of their creep behavior have been thoroughly analyzed. Building upon the foundation of optimizing the intrinsic material properties, this chapter shifts the research focus from the microscale material level to the macroscopic structural design. To systematically investigate the influence of geometric parameters on the overall thermal conductivity of copper micropillar arrays, this work involves fabricating a series of copper micropillar arrays with varying spacing and experimentally measuring their thermal conductivity. The objective is to establish a quantitative relationship between the key structural parameter of pillar spacing and the macroscopic thermal performance, thereby providing both experimental evidence and theoretical guidance for the optimal design of copper micropillar‐based thermal structures.

**FIGURE 13:**
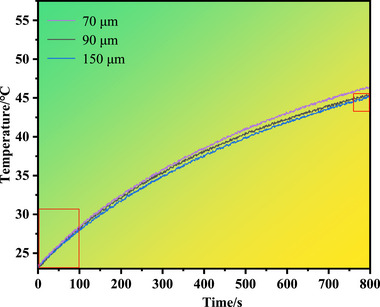
Temperature rise over time of glass substrates with varying copper micropillar array spacings.

Figure 13 illustrates the temperature evolution over time of glass substrates embedded with copper micropillar arrays of varying spacings under identical heat input conditions. Based on the temperature profiles, it is observed that within the initial heating period of 0–100 s, the temperature rise of the glass substrates varies only slightly across different micropillar spacings, as highlighted in the lower‐left rectangle of Figure 13. However, as the heating time extends to 800 s, the temperature rise of the array with a pillar spacing of 70 µm reaches 46.5°C, whereas the arrays with spacings of 90 and 150 µm exhibit nearly identical temperature rises of 45.1°C, as indicated in the right rectangle. This suggests that the 70 µm spacing array, owing to its highest pillar density, forms the most densely packed parallel thermal conduction network, thereby achieving the lowest equivalent thermal resistance and the strongest heat conduction capability, as reflected by the highest temperature rise (46.5°C). As the pillar spacing increases to 90 µm and 150 µm, the reduction in parallel conduction pathways leads to an increase in equivalent thermal resistance and a relative decline in heat conduction performance. Notably, the convergence in temperature rise between the 90 and 150 µm arrays (both at 45.1°C) implies that beyond this spacing range, the internal thermal conduction of the array is no longer the sole limiting factor for the overall thermal performance of the system. Instead, interfacial thermal resistance or the heat dissipation capacity at the top surface may become the new dominant factors, resulting in similar thermal behavior between the two configurations. This finding underscores the importance of comprehensively considering both internal heat conduction and interfacial/external heat dissipation processes in microscale thermal management design, in order to achieve optimal system performance.

However, it should be noted that the experimental conditions in this chapter primarily focus on the analysis of natural convection and heat transfer in a static air environment, without addressing the comprehensive performance evaluation under active cooling conditions. In practical applications, copper micropillar arrays typically serve as the core component of microchannel heat sinks, where their heat dissipation capability depends not only on their intrinsic thermal conduction properties but also on parameters such as the geometric structure of the microchannels, the physical properties of the coolant, and its flow rate and velocity. These factors are interrelated through complex coupling relationships, which collectively determine the thermal management efficiency of the entire system. In our next paper, we will present in detail the thermo‐fluid coupling characteristics of copper micropillar arrays with varying spacings under different flow rates and microchannel configurations, and explore design methodologies for the synergistic optimization of structural and operational parameters.

## 3 Conclusion

In this study, copper micropillar arrays with excellent thermal conductivity were successfully fabricated using the TGV process. We systematically investigated the microstructural evolution and the underlying mechanisms governing creep displacement in copper micropillars under various heat treatment temperatures, deeply analyzed the intrinsic correlation between their viscoplastic behavior and microstructural changes, and experimentally validated the thermal conductivity of micropillar arrays with different spacings. This study not only provides guidance for the fabrication and reliability design of TGV copper micropillar arrays, but also offers valuable insights for the performance optimization of other microscale metallic components by revealing the correlation between “microstructure and creep behavior.” The main conclusions are summarized as follows:

Heat treatment temperature significantly affects the creep performance of copper micropillars. As the temperature increases, the creep displacement first increases and then decreases. After heat treatment at 200°C, the creep displacement reaches its maximum value of 101 nm, accompanied by a distinct transition in creep mechanism. When the heat treatment temperature is raised to 300°C, the creep displacement drops to its minimum of 42 nm. The dynamic evolution of the internal microstructure in copper micropillars serves as the intrinsic driving force for changes in their creep mechanism, creep compliance, and hysteresis spectra. At a heat treatment temperature of 200°C, the micropillars exhibit the smallest activation volume and the lowest dislocation density, indicative of grain boundary sliding creep. In contrast, at 300°C, the micropillars display the largest activation volume and the highest dislocation density. The increased dislocation density enhances the pinning effect on grain boundaries, hindering their relative sliding and thereby promoting a transition from grain boundary sliding creep to dislocation climb‐type creep. The geometric parameter of micropillar spacing significantly influences the macroscopic thermal conduction performance. When the micropillar spacing increases beyond a certain threshold, internal thermal conduction is no longer the sole limiting factor for the overall thermal performance of the system; instead, interfacial thermal resistance and environmental heat dissipation become the dominant bottlenecks. In our next paper, we will present a detailed analysis of the thermo‐fluid coupling characteristics of copper micropillar arrays with varying spacings under different flow rates and microchannel configurations, and explore design methodologies for the synergistic optimization of structural and operational parameters.

## 4 Experiment

### 4.1 Preparation of Micro‐Pillar Thermally Conductive Sample

**FIGURE 14:**
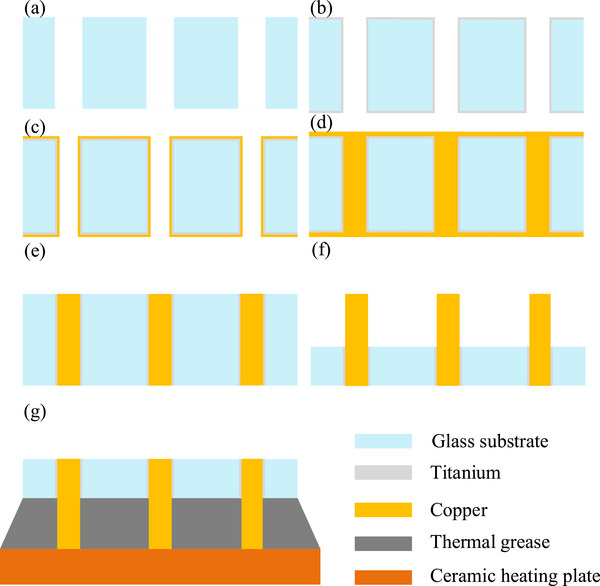
Flow chart of the fabrication process for the micro‐pillar thermally conductive specimen.

This section primarily describes the preparation of thermal conductivity samples of copper micropillar arrays. A glass wafer is patterned with an array of through‐holes, each having a diameter of 50 micrometers and a depth of 300 micrometers (resulting in an aspect ratio of 6:1 for the through‐glass vias). In this study, three types of micropillar arrays with different center‐to‐center pitches were fabricated: 70 µm, 90 µm, and 150 µm. The process flow diagram for preparing the samples used to test the thermal conductivity of the copper micropillar arrays is shown in Figure 14, and the specific steps are described as follows.

- A 300 µm thick glass wafer was prepared and ultrasonically cleaned. It was then subjected to modification using an ultrafast laser with a pulse width of 1000 ps and pulse energy of 12 µJ to induce modified regions in the glass substrate. Subsequently, the wafer was immersed in a proprietary etchant solution to perform through‐hole etching.
- Prior to through‐hole metallization, the glass wafer was again ultrasonically cleaned. A titanium (Ti) film with a thickness of approximately 60 nm was then deposited on the inner walls of the through‐glass vias (TGVs) using pulsed magnetron sputtering, serving as an adhesion layer.
- A copper (Cu) seed layer with a thickness of approximately 1 µm was deposited on the aforementioned surface via pulsed magnetron sputtering.
- The through‐holes were filled using an electroplating process.
- For the heat treatment procedure, the temperature was raised to 100°C, 200°C, or 300°C at 5°C/min. The samples were held at the designated temperature for 30 min and subsequently cooled to ambient conditions in the furnace. After heat treatment, the copper on both sides of the TGV substrate was thinned using a copper etchant, followed by removal through mechanical polishing.
- Using a wet etching process, one side of the TGV substrate was protected with photoresist, while the other side underwent glass etching to expose the copper micropillar array, as is shown in Figure 1d.
- After applying thermal grease to the copper micropillar array prepared in step (f), it was placed onto the upper surface of a ceramic heating plate.

### 4.2 Nanoindentation Creep Experiments

The nanoindentation creep experiments were conducted using a Bruker Nano Indenter TI980 equipped with a Berkovich indenter. The initial indenter calibration was performed using a standard reference sample. Prior to the creep test, the apparatus was stabilized for 5 h to achieve optimal stability and minimize drift. During the nanoindentation creep experiment, a constant load of 80 mN was applied and held for 100 s. The loading strain rate was set at 0.05 s^−^^1^, and the unloading rate was kept the same as the loading rate.

### 4.3 Microstructure Investigation

Thermal performance tests were conducted under a nitrogen atmosphere using a Differential Scanning Calorimetry (DSC3, METTLER TOLEDO). The experiment temperature range was 25–300°C with a heating rate of 5°C/min. The mass changes of the sample during the heating process were observed using a METTLER TOLEDO (Switzerland) thermal analyzer (TGA2) under a nitrogen atmosphere. The tests were performed over a temperature range of 25–300°C at a heating rate of 5°C/min to obtain the TG curves. The copper micropillar array, recrystallized microstructure, and its relationship to the microstructural characteristics of the copper micropillars after heat treatment were investigated using a scanning electron microscope (SEM) equipped with an electron backscatter diffraction (EBSD) probe (EBALSD). Special attention was paid to the grain boundary features within the copper pillars, as this factor may have a profound effect on their creep behavior. The EBSD analysis was conducted at a scanning voltage of 20 kV and a step size of 0.15 µm to ensure a comprehensive characterization of the microstructural features of the copper micropillars. Scanning transmission electron microscopy (STEM)‐high‐angle annular bright field (HAADF) images were acquired using a Talos F200X G2 field emission transmission electron microscope equipped with a Panther STEM detector at 200 kV. TEM samples were prepared using a TESCAN AMBER gallium‐focused ion beam scanning electron microscope (FIB‐SEM Dual Beam).

### 4.4 Evaluation of Thermal Conductance in Micropillar Arrays

A DC power supply (model TDP‐3020 PC) was connected to the leads of a ceramic resistor plate to heat the copper micropillar array sample from the bottom. The input current and voltage were set to 1.5 A and 32 V, respectively. Prior to the experiment, the K‐type thermocouples were calibrated using a comparison method, in which the thermocouples to be calibrated and a standard thermocouple were placed simultaneously in the same temperature field. This calibration was performed to eliminate systematic errors. To measure the surface temperature of the TGV substrate with copper micropillar arrays at different pitches, K‐type thermocouples were attached to the substrate surface using thermal conductive adhesive (Hengan, HY510). The temperature readings were recorded using a 4‐channel temperature measurement instrument (model KLH8004U), which offers an excellent temperature accuracy of 0.1°C.

## Funding

Shenzhen Science and Technology Innovation Bureau (Grant No. KJZD20240903104002004), National Key Research and Development Program (Grant No. 2023YFB4606800), Dongguan Key Research and Development Program (Grant No. 20221200300092).

## Conflicts of Interest

The authors declare no conflict of interest.

## Supporting information




**Supporting File**: advs74326‐sup‐0001‐SuppMat.doc.

## Data Availability

The data that support the findings of this study are available from the corresponding author upon reasonable request.
