# Supplementary material for: Thermally Induced Creep and Viscoelastic Behavior of Copper Micropillar Arrays
Source: Adv Sci (Weinh). 2026 Feb 17;13(22):e19178. doi: 10.1002/advs.202519178 (PMC13088336; doi:10.1002/advs.202519178)
Supplement: Supplementary file 1 — Supporting File: advs74326‐sup‐0001‐SuppMat.doc. [file ADVS-13-e19178-s001.doc]

**Thermally induced creep and viscoelastic behavior of copper micropillar arrays**

*Miao Wang, Jihua Zhang***, Libin Gao, Hongwei Chen, Wenbo Luo, Wenlei Li, Mingcheng Chen, Mengru Li,* *Dongbin Wang,* *Shuang Li, Ting Liu, Xingzhou Cai, Yong Li, Bin Peng, Wanli Zhang*

**Fig. S1.** The load-displacement curves (a) and displacement-time curves (b) of copper micropillars.

**Fig. S2.** Experimental and fitted creep displacement-holding time curve.

**Table S1**

The fitting results of parameters in **Eq. (8)**.

|  | h_0_ | h_1_ | δ_1_ | h_2_ | δ_2_ | μ_0_ | R^2^ |
| --- | --- | --- | --- | --- | --- | --- | --- |
| Room temperature | 1671.57 | 15.92 | 9.72 | 21.78 | 0.68 | 9.26 | 0.99 |
| 100 ℃ | 1830.83 | 22.56 | 10.60 | 29.41 | 0.69 | 5.76 | 0.99 |
| 200 ℃ | 1745.46 | 21.46 | 7.35 | 22.16 | 0.62 | 1.73 | 0.99 |
| 250 ℃ | 1653.30 | 22.12 | 8.19 | 28.70 | 0.51 | 9.89 | 0.99 |
| 300 ℃ | 1710.44 | 18.07 | 9.47 | 18.24 | 0.79 | 16.08 | 0.99 |
